# Supplementary material for: Structural basis for proficient oxidized ribonucleotide insertion in double strand break repair
Source: Nat Commun. 2021 Aug 20;12:5055. doi: 10.1038/s41467-021-24486-x (PMC8379156; doi:10.1038/s41467-021-24486-x)
Supplement: Supplementary file 1 — Supplementary Information [file 41467_2021_24486_MOESM1_ESM.pdf]

SUPPLEMENTARY INFORMATION

Structural Basis for Proficient Oxidized Ribonucleotide Insertion in Double Strand Break Repair

Joonas A. Jamsen[1,*], Akira Sassa[2], Lalith Perera[1], David D. Shock[1], William A. Beard[1] & Samuel H. Wilson[1,*]

[1]Genome Integrity and Structural Biology Laboratory, National Institute of Environmental Health Sciences, National Institutes of Health, Research Triangle Park, NC 27709, USA.

[2]Laboratory of Chromatin Metabolism and Epigenetics, Graduate School of Science, Chiba University, Chiba 263-8522, Japan.

*Correspondence should be addressed to S.H.W. (email: wilson5@niehs.nih.gov) or J.A.J. (email: joonas.jamsen@nih.gov)

This File Contains:

Supplementary Figures 1-6

Supplementary Tables 1-8

Supplementary References

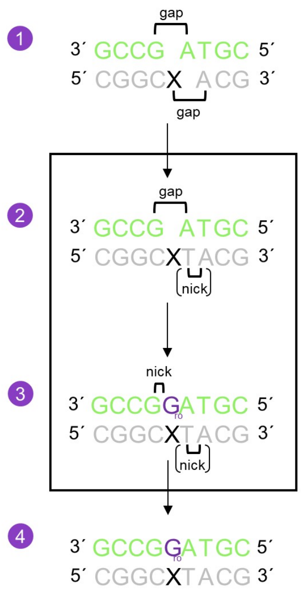

Supplementary Figure 1. Potential substrate DNA configurations in repair of a double strand break involving a single nucleotide gapped intermediate (step 2). 1) Double strand break with single nucleotide gaps in primer (green) and template (grey) strands, 2) gap in template (grey) strand is filled leaving a nick and a single nucleotide gap in the primer (green) strand, 3) 8-oxo-rGTP (G_{ro}, purple) is inserted into the gap in the primer (green) strand leaving nicks in both strands, and 4) nick(s) are ligated generating an intact duplex. Double strand break repair intermediates observed in this study (steps 2 & 3) are indicated with a black box. The nucleotide sequences shown are those employed in time-lapse crystallography (see Methods, black X is A or C). The brackets in intermediates 2) and 3) indicate that nucleotide insertion on a substrate with a nick in the template strand proceeds identically to that on an intact template strand[1].

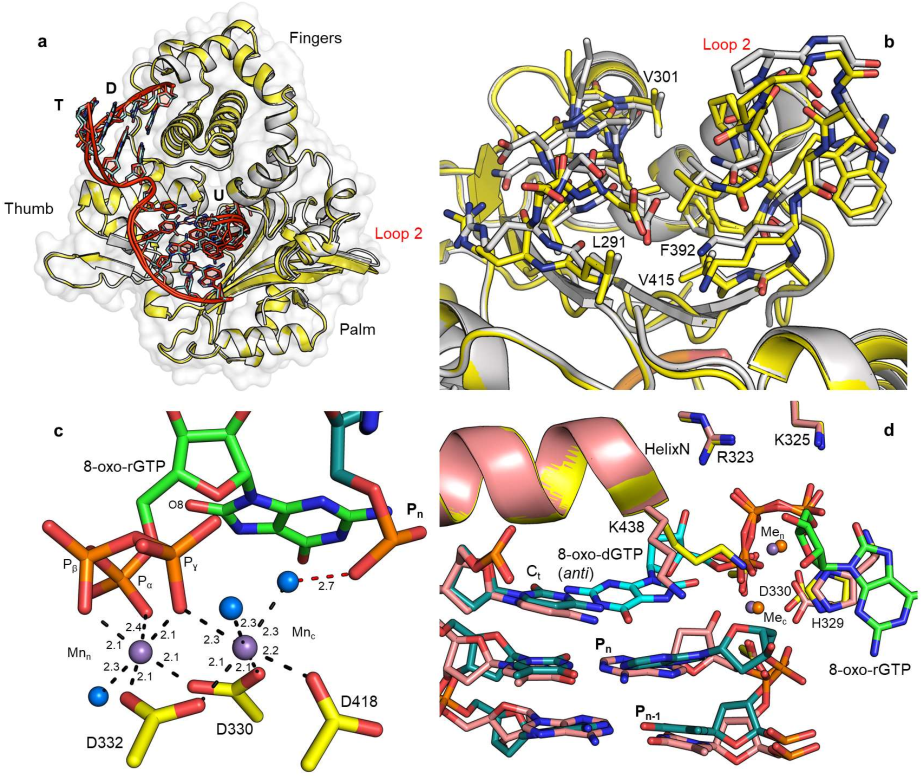

Supplementary Figure 2. Ground state 8-oxo-rGTP ternary complex opposite cytosine. a, Global conformational changes induced by 8-oxo-rGTP. Shown is an overlay of the 8-oxo-rGTP:C_t (yellow) and 8-oxo-dGTP(*anti*):C_t (white; PDB id 7KTA[2]) Ca^{2+}-ground state (GS) ternary complexes. Protein is shown in ribbon representation with atomic volume displayed as a transparent surface. DNA is in cyan (8-oxo-rGTP) or red (8-oxo-dGTP) stick representation. **b**, Close up view of shifts in loop 2 and adjacent regions. Shifted residues are shown in stick representation, other regions in cartoon. **c**, Metal coordination in the Mn^{2+}-ground state 8-oxo-rGTP:C_t ternary complex. P_γ(8-oxo-rGTP) in the unreactive orientation coordinates both Mn_n and Mn_c in the position occupied by P_α in the reacted conformation, while P_β(8-oxo-rGTP) coordinates Mn_n in both orientations. The primer terminus (P_n) adopts a rotated conformation, where O3′ does not coordinate Mn_c. An additional water molecule completes the Mn_c coordination sphere and is stabilized by interactions with P_n (red dashes). Metal coordination (Å) is shown with black dashes. Protein sidechains are in yellow, DNA in cyan, nucleotide in green. Mn^{2+} are the magenta spheres, waters are blue. **d**, Structural overlay of the 8-oxo-dGTP(*anti*):C_t (protein in salmon, nucleotide in light cyan; PDB id 7KTA[2]) Ca^{2+}-GS and 8-oxo-rGTP:C_t (protein in yellow, DNA in dark cyan, nucleotide in green) Mn^{2+}-GS ternary complexes. Lys438 stabilizes the unreactive orientation, while adjustments in the downstream and template strands accommodate the inverted primer terminus (P_n). Ca^{2+} atoms are shown as orange spheres.

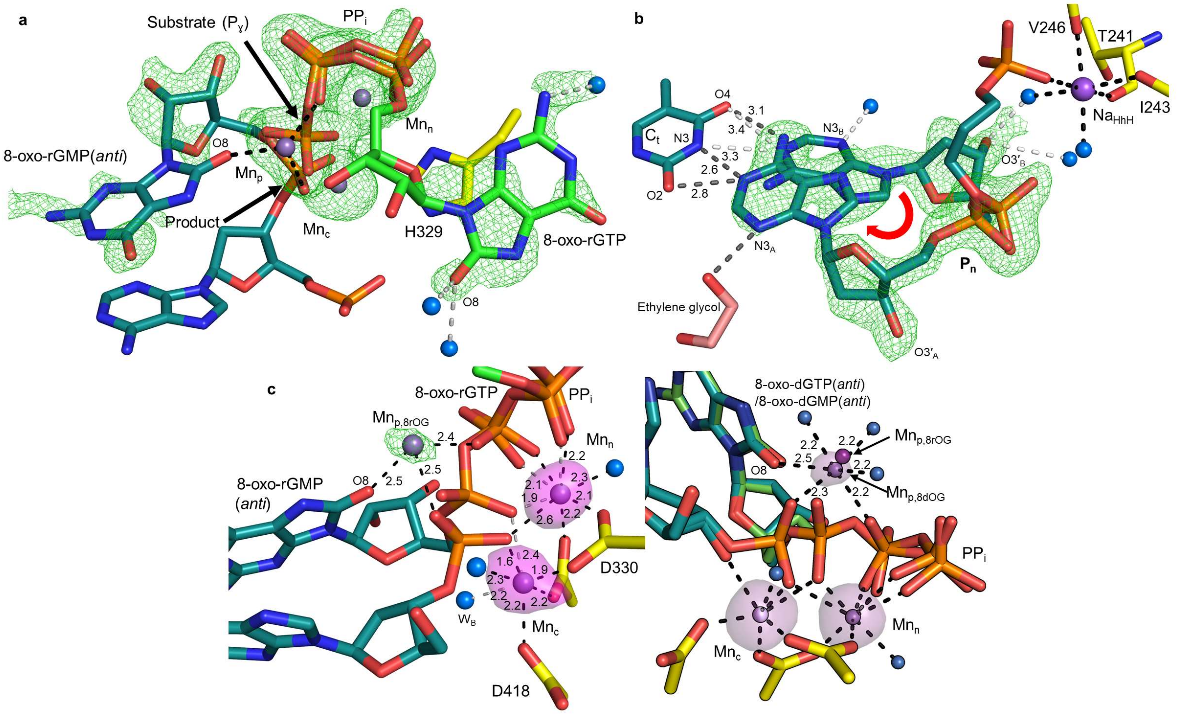

Supplementary Figure 3. Reaction state 8-oxo-rGTP(*anti*):C_t ternary complex. a, 8-oxo-rGTP alters conformation to allow nucleotide insertion. Diffuse density for the base and ribose sugar of 8-oxo-rGMP(*anti*) is observed in the reacted conformation, while density is present for the base but absent for the ribose sugar of 8-oxo-rGTP in the unreactive conformation. The alternate primer terminus conformation was omitted for clarity. $Mn_{p,8rOG}$ coordination is shown with black dashes, hydrogen bonding is shown with grey dashes. DNA is shown in cyan stick representation, sidechains in yellow and nucleotide in green. Mn^{2+} is shown as a magenta sphere and waters are in blue. Simulated annealing omit (F_o-F_c) density (green mesh) is contoured at 2.5 σ. **b**, Conformational change of the primer terminal nucleotide enables 8-oxo-rGTP(*anti*):C_t insertion. The view shown is ~180° compared to panel **a**. Unique interactions stabilize the reacted and unreactive primer termini. Hydrogen bonding is shown as dark grey and white dashed lines for the reacted (conformation A) and unreactive (conformation B) primer termini, respectively. Density consistent with catalysis but lacking bond formation was not observed. Simulated annealing omit (F_o-F_c) density shown as a green mesh is contoured at 3 σ. **c**, Close-up view of metal coordination in the ribo- (left) and deoxy-8-oxo-GTP/GMP (right) active sites opposite C_t. Alternate conformations of the primer terminus and 8-oxo-rGTP were truncated for clarity (left panel). The panel on the right displays $Mn_{p,8rOG}$ overlaid on the Mn^{2+}:8-oxo-dGTP(*anti*)/dGMP(*anti*):C_t reaction state ternary complex (PDB id 7KTB[2]). Metal coordination (Å) is shown with black dashes. Anomalous density (magenta mesh) is contoured at 5 σ, simulated annealing (F_o-F_c) omit density (green mesh near $Mn_{p,8rOG}$) is contoured at 3 σ.

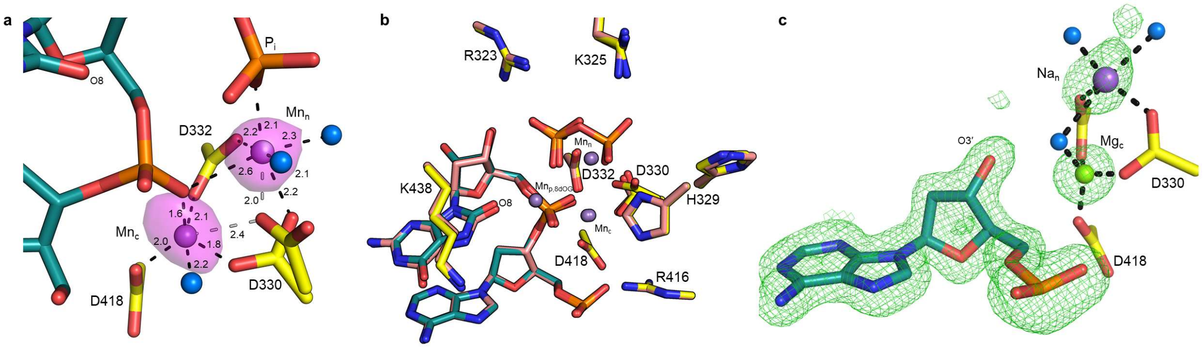

Supplementary Figure 4. 8-oxo-rGMP(*anti*):C$_t$ product complex. a, Close up view of active site metal coordination in the Mn^{2+}-product complex of the 8-oxo-rGTP(*anti*):C$_t$ insertion. Short coordination distances (black dashes, Å) between catalytic metal (Mn$_c$) and incorporated phosphate oxygen as well as Asp330 are observed. Coordination (Å) for an alternate ~90° rotated conformation of Asp330 is shown as light gray dashes. Anomalous density is shown as a (purple) surface contoured at 5 σ. Protein sidechains are shown in yellow stick representation, DNA in cyan. Mn^{2+} atoms are the purple spheres and water molecules are shown as blue spheres. **b**, Overlay of the Mn^{2+} ribo- and deoxy-8-oxo-GMP(*anti*):C$_t$ (PDB id 7KTC[2]) product complexes. The active sites are identical apart from the product metal in the deoxy- structure that is absent in the ribo-8-oxo-GMP product complex. P2 of PP$_i$ (former P$_\gamma$ of 8-oxo-rGTP) has dissociated in the latter and displays a rotated Asp330. Protein sidechains are shown in yellow (ribo) or salmon (deoxy), DNA in cyan (ribo) or salmon (deoxy). **c**, Active site of the extended (2160 min) Mg^{2+} soak of the 8-oxo-rGTP:C$_t$ insertion. The active site is vacant and density for either incoming or incorporated 8-oxo-rGTP is absent. Metal coordination (black dashes) suggests Mg^{2+} occupies the catalytic metal site (Mg$_c$), while Na^+ is bound in the nucleotide metal site (Na$_n$). Mg$_c$ is shown as a green sphere, Na^+ as a large purple sphere. Simulated annealing (F_o-F_c) omit density shown as a green mesh is contoured at 3 σ with a carve radius of 4 Å.

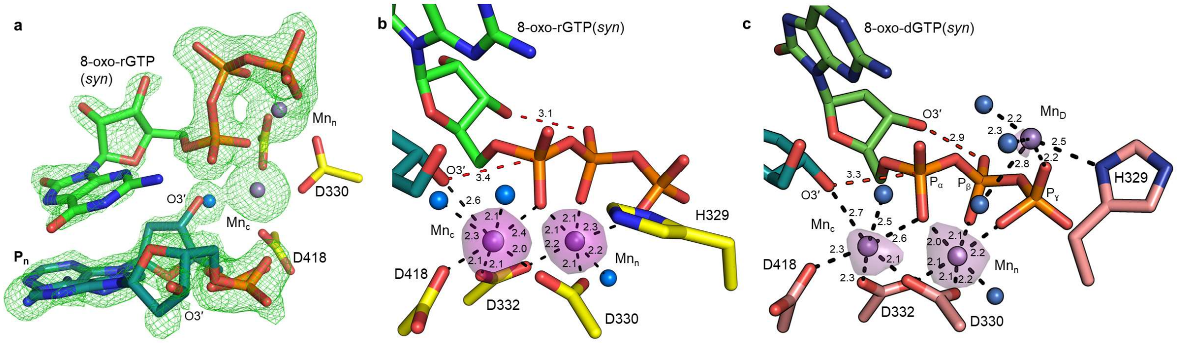

Supplementary Figure 5. Mn^{2+}-ground state ternary complex opposite adenine. a, Mn^{2+}-ground state 8-oxo-rGTP(*syn*):A_t ternary complex. 8-oxo-rGTP is shown in green stick representation, DNA in cyan, sidechains in yellow. Mn^{2+} atoms are shown as magenta spheres, water is a blue sphere. Simulated annealing omit (F_o-F_c) density (green mesh) shown is contoured at 3 σ. **b**, Close up view (~90° rotation of panel **a**) of metal coordination in the Mn^{2+}-ground state 8-oxo-rGTP(*syn*):A_t ternary complex. His329 adopts a position proximal to Asp330 to stabilize P_γ. Coordination distances (Å) are labeled and shown with black dashes, red dashes indicate key distances (Å). Anomalous density (purple surface) is contoured at 5 σ. The alternate conformation of the primer terminal nucleotide (P_n) was omitted for clarity. **c**, An additional metal (Mn_D) coordinates P_γ in the 15 min Mn^{2+} soak of the 8-oxo-dGTP(*syn*):A_t insertion. His329 has altered conformation from a position proximal to Asp330 and now coordinates Mn_D. Protein sidechains are shown in salmon stick representation, 8-oxo-dGTP in dark green, DNA in cyan. Anomalous density is shown as a magenta surface contoured at 5 σ.

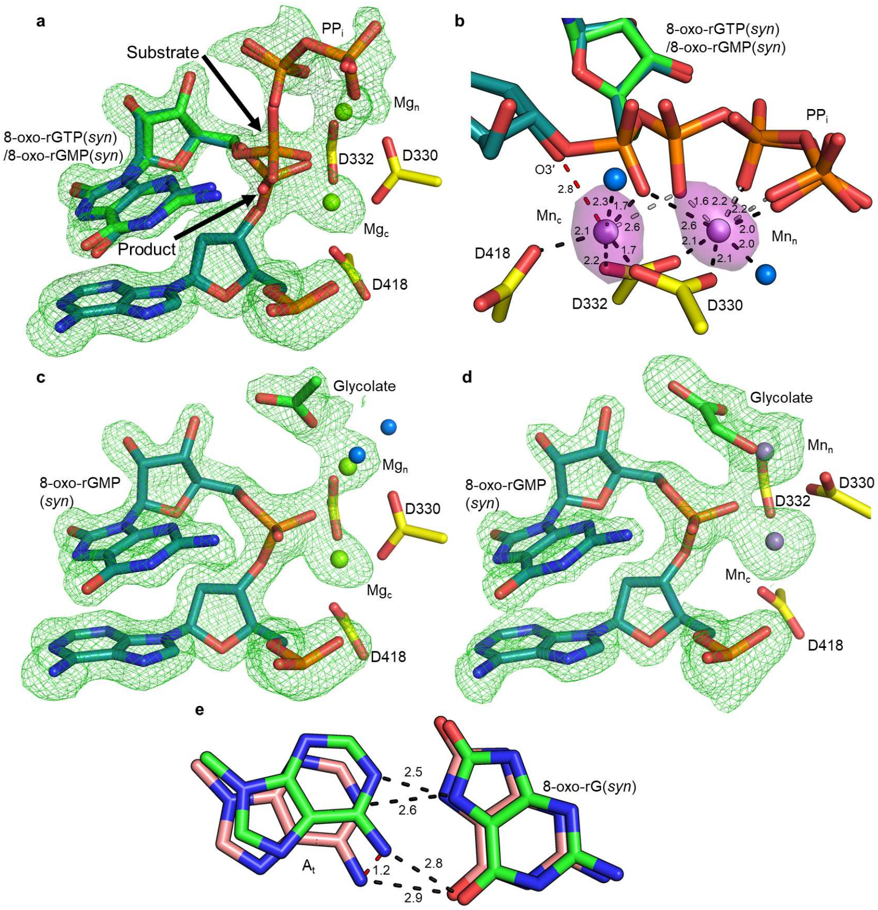

Supplementary Figure 6. 8-oxo-rGTP(*syn*) insertion opposite adenine. a, Mg^{2+}-reaction state 8-oxo-rGMP(*syn*)/rGTP(*syn*):A_t ternary complex. Density is unusually weak for the major groove oxygen of P_{n+1} and the P_α-P_β bond, yet consistently strong for P_α and P1 of PP_i (former P_β of 8-oxo-rGTP), across all Mg^{2+} partial catalysis complexes. Mg^{2+} atoms are shown as green spheres, protein sidechains in yellow stick representation, DNA in cyan, 8-oxo-rGTP(*syn*) in green. Green mesh (simulated annealing (F_o-F_c) omit density) is contoured at 3 σ. **b**, Metal coordination in the Mn^{2+}:8-oxo-rGMP(*syn*)/rGTP(*syn*):A_t reaction state (RS) complex. Coordination (Å) is shown with black dashes for the product and white dashes for alternate coordination by substrate. Key distance (Å) is shown with a red dashed line. Mn^{2+} atoms and water molecules are shown as purple and blue spheres, respectively. Anomalous density (purple surface) is contoured at 5 σ. **c**, 8-oxo-rGMP(*syn*):A_t product complex after a 120 min soak in an Mg^{2+}-containing cryo-solution. A partial glycolate (green) was modeled into the density vacated by PP_i. **d**, Mn^{2+}:8-oxo-rGMP(*syn*):A_t product complex after a 960 min soak. **e**, Nascent 8-oxo-rGMP(*syn*):A_t base pair in the Mg^{2+}-product (salmon) and extended soak (green) complexes. Hydrogen bonding (Å) is shown as black dashed lines, key distance (Å) is shown with a red dashed line.

Supplementary Table 1. Kinetic parameters for pol μ nucleotide insertion in the presence of Mg^{2+}.

dNTP	Template	K_m, μM	k_{cat}, min^{-1}	k_{cat}/K_m, $μM^{-1}$ min^{-1}
dGTP[1]	dC	3.48 ± 0.31	5.70 ± 0.21	1.64 ± 0.21
	dA	62.4 ± 11.6	0.07 ± 0.01	0.0011 ± 0.0002
rGTP	dC	6.11 ± 1.11	8.30 ± 0.47	1.36 ± 0.26
	dA	58.8 ± 16.9	0.015 ± 0.001	0.00026 ± 0.0001
8-oxo-dGTP[1]	dC	141 ± 14	1.35 ± 0.04	0.010 ± 0.001
	dA	31.3 ± 1.5	8.20 ± 0.16	0.26 ± 0.02
8-oxo-rGTP	dC	241 ± 16	0.051 ± 0.001	0.00021 ± 0.00002
	dA	69.9 ± 5.5	0.76 ± 0.02	0.011 ± 0.001

[1]Jamsen J. *et al.*[2]

*The values reported are the mean ± S.E. of three independent measurements.

Supplementary Table 2. Kinetic parameters for pol μ nucleotide insertion in the presence of Mn^{2+}.

dNTP	Template	K_m, μM	k_{cat}, min^{-1}	k_{cat}/K_m, μM^{-1} min^{-1}
dGTP[1]	dC	0.006 ± 0.001	0.16 ± 0.01	26.9 ± 4.5
	dA	21.3 ± 2.3	2.98 ± 0.09	0.14 ± 0.02
rGTP	dC	0.031 ± 0.004	0.77 ± 0.04	24.8 ± 3.5
	dA	22.2 ± 2.8	0.70 ± 0.03	0.032 ± 0.001
8-oxo-dGTP[1]	dC	5.03 ± 1.19	5.92 ± 0.41	1.18 ± 0.36
	dA	7.80 ± 2.22	5.14 ± 0.42	0.66 ± 0.24
8-oxo-rGTP	dC	30.4 ± 2.60	2.36 ± 0.06	0.078 ± 0.007
	dA	12.2 ± 2.2	8.92 ± 0.45	0.73 ± 0.14

[1]Jamsen J. *et al.*[2]

*The values reported are the mean ± S.E. of three independent measurements.

Supplementary Table 3. Crystallographic statistics for structures opposite C_t.

	8rOG(*anti*):C_t Ca^{2+}, 120 min	8rOG(*anti*):C_t Mn^{2+}, 15 min	8rOG(*anti*):C_t Mn^{2+}, 960 min	8rOG(*anti*):C_t Mn^{2+}, 2160 min	8rOG(*anti*):C_t Mg^{2+}, 2160 min
**PDB ID**	**6VF8**	**6VFA**	**6VFB**	**6VFC**	**6VF9**
**Data Collection**					
Space Group	$P2_12_12_1$	$P2_12_12_1$	$P2_12_12_1$	$P2_12_12_1$	$P2_12_12_1$
Cell Dimensions					
a, b, c (Å)	60.101	60.079	60.198	60.333	60.200
	62.271	62.215	62.334	62.440	62.130
	118.637	118.925	118.756	119.336	118.533
α, β, γ (°)	90, 90, 90	90, 90, 90	90, 90, 90	90, 90, 90	90, 90, 90
Resolution (Å)[1]	50 – 1.70	50 – 1.76	50 – 1.55	50 – 1.59	50 – 1.56
	(1.76 – 1.70)	(1.82 – 1.76)	(1.60 – 1.55)	(1.65 – 1.59)	(1.62 – 1.56)
R_{sym} or R_{merge}[1]	11.1 (76.3)	10.5 (84.7)	10.1 (62.2)	5.0 (95.2)	7.6 (91.1)
$I/\sigma I$[1]	19.0 (2.0)	13.6 (2.0)	17.1 (2.1)	27.0 (2.1)	19.6 (2.1)
Completeness (%)[1]	99.9 (100.0)	99.1 (99.7)	99.8 (99.0)	95.8 (97.2)	99.0 (99.7)
Redundancy[1]	6.6 (5.7)	5.4 (4.8)	5.7 (3.4)	5.6 (5.6)	6.7 (5.9)
No. Unique Refl.[1]	49,850	45,213	66,099	61,513	64,138
**Refinement**					
RS : PS (%)	100 : 0	100 : 0	50 : 50	0 : >80	–
A site occ (%)	100 Ca^{2+}	100 Mn^{2+}	100 Mn^{2+}	90 Mn^{2+}	70 Mg^{2+}
B site occ (%)	100 Ca^{2+}	100 Mn^{2+}	100 Mn^{2+}	100 Mn^{2+}	70 Na^{+}
C site occ (%)	–	–	25 Mn^{2+}	–	–
D site occ (%)	–	–	–	–	–
Resolution (Å)	35 – 1.70	35 – 1.76	35 – 1.55	35 – 1.59	35 – 1.56
No. Reflections	49,447	44,727	65,893	58,828	63,419
R_{work} / R_{free}	0.17 / 0.19	0.18 / 0.20	0.17 / 0.18	0.17 / 0.19	0.17 / 0.18
No. atoms					
Protein / DNA	2611 / 380	2599 / 342	2600 / 401	2600 / 380	2671 / 342
dNTP / PP_i / Metal	15 / 0 / 3	33 / 0 / 9	33 / 9 / 8	0 / 0 / 9	0 / 0 / 3
Water / Ligands	312 / 32	304 / 56	302 / 95	322 / 63	356 / 26
B-factors					
Protein / DNA	27.3 / 24.8	29.3 / 27.1	29.6 / 26.8	27.5 / 24.0	26.2 / 21.7
Me_A / Lig_A[2]	16.8 / 23.5	19.4 / 19.7	22.9 / 23.0	20.6 / 22.0	26.2 / 34.9
Me_B / Lig_B[2]	18.0 / 22.5	18.4 / 19.2	19.3 / 21.4	20.5 / 21.6	33.2 / 34.3
Me_C / Lig_C[2]	– / –	– / –	35.2 / 23.5	– / –	– / –
Water / Ligands[3]	35.5 / 38.0	38.4 / 45.5	40.4 / 34.9	37.0 / 36.0	35.5 / 39.7
Wilson B	22.8	23.5	21.8	23.3	21.0
R.M.S Deviations					
Bond Lengths (Å)	0.006	0.006	0.012	0.010	0.008
Bond Angles (°)	0.935	0.898	1.249	1.143	1.023

[1]Data in the highest resolution shell is shown in the parenthesis.

[2]B-factors for the catalytic metal (Me_A), nucleotide metal (Me_B), product metal (Me_C), and surrounding ligands (Lig_A, Lig_B and Lig_C) were obtained using Check My Metals (CMM; https://csgid.org/csgid/metal_sites).

[3]Overall B-factor for ligands.

Supplementary Table 4. Crystallographic statistics for structures opposite A_t in the presence of Ca^{2+} or Mn^{2+}.

	8rOG(*syn*):A_t Ca^{2+}, 120 min	8rOG(*syn*):A_t Mn^{2+}, 15 min	8dOG(*syn*):A_t Mn^{2+}, 15 min	8rOG(*syn*):A_t Mn^{2+}, 30 min	8rOG(*syn*):A_t Mn^{2+}, 120 min	8rOG(*syn*):A_t Mn^{2+}, 960 min
**PDB ID**	**6VEZ**	**6VF3**	**6VF7**	**6VF4**	**6VF5**	**6VF6**
**Data Collection**						
Space Group	$P2_12_12_1$	$P2_12_12_1$	$P2_12_12_1$	$P2_12_12_1$	$P2_12_12_1$	$P2_12_12_1$
Cell Dimensions						
a, b, c (Å)	59.966	60.198	60.159	60.085	59.982	60.263
	68.481	62.385	68.927	68.757	68.718	62.333
	110.194	118.580	110.446	110.531	110.413	118.847
α, β, γ (°)	90, 90, 90	90, 90, 90	90, 90, 90	90, 90, 90	90, 90, 90	90, 90, 90
Resolution (Å)[1]	50 – 1.88	50 – 1.52	50 – 1.87	50 – 1.75	50 – 1.60	50 – 1.69
	(1.94 – 1.88)	(1.58 – 1.52)	(1.94 – 1.87)	(1.81 – 1.75)	(1.66 – 1.60)	(1.75 – 1.69)
R_{sym} or R_{merge}[1]	6.7 (75.2)	5.5 (82.6)	9.3 (93.0)	6.2 (97.0)	7.3 (87.7)	8.1 (99.3)
$I/\sigma I$[1]	26.7 (2.2)	26.1 (2.2)	17.7 (2.1)	28.1 (2.3)	18.5 (2.0)	21.4 (2.3)
Completeness (%)[1]	99.9 (99.9)	99.1 (98.9)	99.5 (99.8)	99.7 (99.7)	99.1 (98.1)	99.8 (99.8)
Redundancy[1]	7.9 (4.5)	6.3 (6.5)	5.1 (4.8)	6.5 (5.8)	5.1 (4.5)	6.5 (5.9)
No. Unique Refl.[1]	37,879	69,288	38,701	46,976	60,947	50,916
**Refinement**						
RS : PS (%)	100 : 0	100 : 0	100 : 0	60 : 40	0 : 100	0 : 100
A site occ (%)	100 Ca^{2+}	100 Mn^{2+}	100 Mn^{2+}	100 Mn^{2+}	100 Mn^{2+}	100 Mn^{2+}
B site occ (%)	100 Ca^{2+}	100 Mn^{2+}	100 Mn^{2+}	100 Mn^{2+}	100 Mn^{2+}	100 Mn^{2+}
C site occ (%)	–	–	–	–	–	–
D site occ (%)	–	–	50 Mn^{2+}	–	–	–
Resolution (Å)	25 – 1.88	35 – 1.52	35 – 1.87	35 – 1.75	40 – 1.60	35 – 1.69
No. Reflections	37,563	68,611	38,457	46,761	60,306	50,726
R_{work} / R_{free}	0.17 / 0.21	0.16 / 0.18	0.17 / 0.20	0.17 / 0.19	0.17 / 0.19	0.17 / 0.19
No. atoms						
Protein / DNA	2721 / 385	2611 / 424	2642 / 385	2644 / 406	2637 / 385	2593 / 344
dNTP / PP_i / Metal	33 / 0 / 4	33 / 0 / 9	32 / 0 / 9	33 / 9 / 6	0 / 0 / 8	0 / 0 / 9
Water / Ligands	343 / 53	334 / 59	327 / 59	287 / 88	352 / 57	307 / 52
B-factors						
Protein / DNA	27.7 / 26.3	27.5 / 23.8	30.3 / 30.1	31.2 / 30.2	25.9 / 25.3	29.2 / 23.9
Me_A / Lig_A[2]	23.6 / 34.7	18.4 / 21.6	26.9 / 20.7	22.6 / 23.3	15.8 / 15.2	18.5 / 21.9
Me_B / Lig_B[2]	26.5 / 35.2	18.3 / 20.6	18.7 / 20.3	20.7 / 22.5	14.4 / 15.1	18.4 / 21.3
Me_D / Lig_D[2]	– / –	– / –	29.9 / 31.9	– / –	– / –	– / –
Water / Ligands[3]	35.8 / 46.9	37.6 / 38.7	36.3 / 26.6	38.3 / 31.1	34.5 / 25.0	38.1 / 35.0
Wilson B	26.1	21.7	25.5	26.7	21.1	22.7
R.M.S Deviations						
Bond Lengths (Å)	0.016	0.014	0.010	0.013	0.008	0.013
Bond Angles (°)	1.222	1.421	0.832	1.145	0.988	1.192

[1]Data in the highest resolution shell is shown in the parenthesis.

[2]B-factors for the catalytic metal (Me_A), nucleotide metal (Me_B), product metal (Me_C), and surrounding ligands (Lig_A, Lig_B and Lig_C) were obtained using Check My Metals (CMM; https://csgid.org/csgid/metal_sites).

[3]Overall B-factor for ligands.

Supplementary Table 5. Crystallographic statistics for structures opposite A_t in the presence of Mg^{2+}.

	8rOG(*syn*):A_t Mg^{2+}, 30 min	8rOG(*syn*):A_t Mg^{2+}, 120 min	8rOG(*syn*):A_t Mg^{2+}, 960 min
**PDB ID**	**6VF0**	**6VF1**	**6VF2**
**Data Collection**			
Space Group	$P2_12_12_1$	$P2_12_12_1$	$P2_12_12_1$
Cell Dimensions			
a, b, c (Å)	59.982	60.044	60.278
	68.437	68.415	62.297
	110.615	111.070	118.573
α, β, γ (°)	90, 90, 90	90, 90, 90	90, 90, 90
Resolution (Å)[1]	50 – 1.57	50 – 1.68	50 – 1.60
	(1.63 – 1.57)	(1.74 – 1.68)	(1.66 – 1.60)
R_{sym} or R_{merge}[1]	10.5 (97.1)	7.5 (99.6)	7.2 (91.7)
$I/\sigma I$[1]	16.1 (2.0)	19.3 (2.4)	23.1 (2.0)
Completeness (%)[1]	99.8 (99.6)	99.3 (99.5)	100.0 (99.9)
Redundancy[1]	6.6 (6.2)	4.6 (4.3)	7.1 (7.0)
No. Unique Refl.[1]	63,656	52,951	59,703
**Refinement**			
RS : PS (%)	60 : 40	0 : 100	0 : 100
A site occ (%)	100 Mg^{2+}	70 Mg^{2+}	60 Mg^{2+}
B site occ (%)	100 Mg^{2+}	90 Mg^{2+}	50 Na^{+}
C site occ (%)	–	–	–
D site occ (%)	–	–	–
Resolution (Å)	35 – 1.57	35 – 1.68	35 – 1.60
No. Reflections	63,471	52,507	59,597
R_{work} / R_{free}	0.16 / 0.19	0.16 / 0.19	0.17 / 0.19
No. atoms			
Protein / DNA	2714 / 406	2697 / 385	2660 / 344
dNTP / PP_i / Metal	33 / 5 / 4	0 / 0 / 4	0 / 0 / 3
Water / Ligands	388 / 89	370 / 43	316 / 52
B-factors			
Protein / DNA	28.6 / 27.1	22.7 / 19.8	29.0 / 24.2
Me_A / Lig_A[2]	26.1 / 43.0	21.0 / 35.2	20.4 / 36.4
Me_B / Lig_B[2]	29.2 / 43.9	29.2 / 33.5	35.2 / 36.9
Water / Ligands[3]	39.0 / 43.8	34.4 / 31.8	38.2 / 38.3
Wilson B	22.2	18.3	22.6
R.M.S Deviations			
Bond Lengths (Å)	0.012	0.013	0.010
Bond Angles (°)	1.195	1.203	1.141

[1]Data in the highest resolution shell is shown in the parenthesis.

[2]B-factors for the catalytic metal (Me_A), nucleotide metal (Me_B), product metal (Me_C), and surrounding ligands (Lig_A, Lig_B and Lig_C) were obtained using Check My Metals (CMM; https://csgid.org/csgid/metal_sites).

[3]Overall B-factor for ligands.

Supplementary Table 6. Selected residue interaction energies in the unreactive or reacted conformations from molecular dynamics analysis.

Residue	Unreactive, kcal mol^{-1}	Reacted, kcal mol^{-1}
E161	-12.02 ± 1.40	0.20 ± 0.03
K187	-4.76 ± 1.37	-0.32 ± 0.07
L317	-0.95 ± 0.14	-0.75 ± 0.16
T318	-2.06 ± 0.25	-2.23 ± 0.48
G319	-10.80 ± 0.82	-10.18 ± 1.30
G320	-8.70 ± 1.14	-6.60 ± 1.53
F321	1.55 ± 0.36	0.81 ± 0.32
R322	-1.49 ± 0.24	-1.15 ± 0.31
R323	-25.53 ± 3.41	-23.62 ± 3.71
G324	-0.20 ± 0.11	-0.07 ± 0.09
K325	-34.50 ± 2.85	-5.19 ± 5.53
G328	-4.86 ± 0.56	-2.35 ± 1.44
H329	-2.88 ± 0.97	-2.45 ± 1.69
D330	37.83 ± 2.78	33.68 ± 3.24
D332	38.98 ± 2.77	39.66 ± 3.53
R416	-4.93 ± 0.88	-4.46 ± 0.72
V417	-1.11 ± 0.19	-1.17 ± 0.25
D418	7.19 ± 0.86	10.21 ± 1.24
G433	-0.23 ± 0.10	0.00 ± 0.37
W434	0.76 ± 0.29	-4.35 ± 0.84
G436	-1.38 ± 0.28	-2.53 ± 0.47
S437	-1.60 ± 0.36	-2.96 ± 0.62
K438	-17.30 ± 7.71	-6.66 ± 1.79
Q441	-0.49 ± 0.15	-9.79 ± 1.17
R445	-0.21 ± 0.03	2.10 ± 0.68

Supplementary Table 7. Selected DNA interaction energies in the unreactive or reacted conformations from molecular dynamics analysis.

Residue	Unreactive, kcal mol^{-1}	Reacted, kcal mol^{-1}
C_t	0.14 $\pm$ 0.03	-14.96 $\pm$ 1.35
T_{n-1}	0.06 $\pm$ 0.02	-1.30 $\pm$ 0.44
P_{n-1}	1.90 $\pm$ 0.34	1.42 $\pm$ 0.23
P_n	4.63 $\pm$ 0.81	-9.56 $\pm$ 2.00

Supplementary Table 8. Sequences of oligonucleotides used in this study.

Experiment	Oligonucleotide	Sequence (5'-3'), template base underlined
Kinetics	Template A	GACGTCGACTACGCG A CATGCCTAGGG GCCCATG
Kinetics	Template C	GACGTCGACTACGCG C CATGCCTAGGG GCCCATG
Kinetics	Upstream	[Cy3]-GTCAGACTGACGTA
Kinetics	Downstream	pGCCGGACGACGGAG
Crystallography	Template A	CGGC A TACG
Crystallography	Template C	CGGC C TACG
Crystallography	Upstream	CGTA
Crystallography	Downstream	pGCCG

Supplementary References

1 Kaminski, A. M. *et al.* Structural snapshots of human DNA polymerase mu engaged on a DNA double-strand break. *Nat Commun* **11**, 4784, doi:10.1038/s41467-020-18506-5 (2020).
2 Jamsen, J. A., Sassa, A., Shock, D. D., Beard, W. A. & Wilson, S. H. Watching a double strand break repair polymerase insert a pro-mutagenic oxidized nucleotide. *Nat Commun* **12**, 2059, doi:10.1038/s41467-021-21354-6 (2021).
